# Global and Regional Left Ventricular Contractile Impairment in Patients with Wolff-Parkinson-White Syndrome

**Published:** 2009-07-01

**Authors:** Luis Afonso, Jyotiranjan Pradhan, Vikas Veeranna, Ashutosh Niraj, Sony Jacob

**Affiliations:** Division of Cardiology/Electrophysiology, Wayne State University, MI, USA

**Keywords:** Wolff-Parkinson-White Syndrome, Strain imaging, regional contractile abnormalities

## Abstract

**Background:**

To assess regional systolic function and global contractile function in patients with WPW Syndrome.

**Method:**

Eleven cases with manifest Wolff-Parkinson-White (WPW) syndrome in sinus rhythm were compared to 11 age matched controls. 2D strain analysis was performed and peak segmental radial strain (pRS) values obtained from basal ventricular parasternal short-axis images (70 ± 5 frames/sec) using a dedicated software package. Heterogeneity of radial strain pattern in six circumferential basal left ventricular segments was measured in terms of standard deviations of peak RS (SD^pRS^) or range (difference between maximum and minimum peak RS i.e. Range^pRS^). Spectral Doppler (continuous wave) measurements were acquired through the left ventricular outflow tract to determine Pre Ejection Period (PEP), Left Ventricular Ejection Time (LVET) and measures of left ventricular systolic performance.

**Results:**

LV segmental radial strain was profoundly heterogeneous in WPW cases in contrast to fairly homogenous strain pattern in normal subjects. Wide SD^pRS^ values 17.5 ± 8.9 vs 3.3 ± 1.4, p<0.001 and Range^pRS^  42.7 ± 20.8 vs.8.5 ± 3.6 ,  p<0.001 were observed among WPW and healthy subjects respectively. PEP (132.4 ± 14.7 vs 4.7 ± 0.5ms, p<0.001) and corrected PEP (76.1 ± 8.0 vs 2.7 ± 0.4ms, p<0.001) were significantly longer in WPW patients compared to controls. The PEP/LVET ratio was also significantly greater in WPW cohort (0.49 ± 0.04 vs. 0.28 ± 0.05, p <0.001) suggesting global systolic dysfunction.

**Conclusions:**

Patients with manifest preexcitation (predominantly those with right-sided pathways) have regional and global contractile dysfunction resulting from aberrant impulse propagation inherent to the preexcited state.

## Introduction

Abnormal electrical activation of the accessory pathway results in characteristic electrocardiographic abnormalities and clinical symptoms manifesting as WPW syndrome [1].  Data on the effects of abnormal ventricular activation on regional contractile patterns of the ventricles in patients with the WPW are  limited to the description of wall motion abnormalities [2-5]. However, wall motion abnormalities are not necessarily  always accompanied by perturbations of contractility [6].

Traditionally, regional left ventricular function has been evaluated using visual wall-motion analysis as well as by other quantitative methods such as integrated backscatter, automatic border detection and tissue Doppler imaging (TDI) [7-9]. However, while velocities obtained by TDI reflect local myofiber shortening, they may also be modulated in part, by motion of and tethering of adjacent segments. Two dimensional strain  is a novel technique that allows the rapid and reproducible quantification of regional contractility from standard black and white two dimensional images  [10,11].

It has recently become evident that chronic right ventricular apical  pacing induced dyssynchrony leads to  deleterious effects on left ventricular systolic function  [12,13].  Accessory bypass tract conduction likewise, leads to an altered sequence of ventricular activation and assorted dyssynergy  patterns that have been extensively studied  [1-5]; whether or not  the resultant dyssynergy influences systolic performance however, is unclear. Measurement of systolic time intervals is a valuable, noninvasive technique to assess left ventricular performance. The  PEP/LVET ratio has been shown to be a very sensitive measure of global systolic ventricular  performance   [14-16].

To the best of our knowledge, no data on the prevalence of left ventricular dysfunction in patients with accessory pathway conduction exists in the literature. We sought to characterize regional left ventricular function in patients with manifest WPW syndrome by examining radial myocardial strain patterns. We also attempted to investigate the presence of global dysfunction using systolic time intervals.

## Methods

### Study population

The study included 11 cases with manifest pre-excitation on surface ECG and an equal number of age-matched controls (Table 1).  None of the patients had diabetes, pathologic valvular disease, bundle branch block, prior infarction, coronary artery disease, hypertension, pericardial disease or active arrhythmias. The University Institutional Review Board approved the study protocol and written informed consent was obtained from all subjects.

### Accessory Bypass tract localization (surface ECG)

All accessory pathways were localized using the algorithm proposed by Milstein and colleagues [17] on a standard 12-lead normal sinus ECG.

### Echocardiography

#### Spectral Doppler Analysis and Systolic Time Intervals

**Figure 1:**
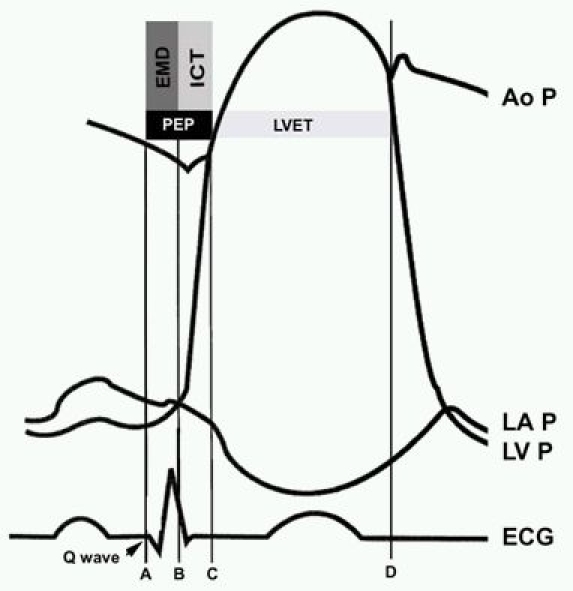
Schematic representation of the components of the pre-ejection period . The pre-ejection period (PEP)(A-C) has two components: the Electromechanical Delay(EMD): the time from the Q wave to the left atrioventricular pressure crossover point; (A-B), and the isovolumic contraction time (ICT) that corresponds to the rapid rise of the left ventricular pressure up to the level of the diastolic aortic pressure (B-C).LVET: Left ventricular Ejection time is the time period from the opening of the Aortic valve to the closure of the Aortic valve (C-D). Ao P- Aortic pressure tracing, LVP- left ventricular pressure tracing and LAP- left atrial pressure tracing, and ECG electrocardiogram

Continuous wave Doppler measurements were acquired through the left ventricular outflow tract. PEP was defined as the time from the beginning of the QRS complex to the onset of ventricular ejection (opening click). LVET was measured from the onset of ventricular ejection (opening click) to the closure of the aortic valve (closure click) (Figure 1). Corrected systolic time intervals for heart rates (cPEP or cLVET) were calculated by using standard Bazett's formula.

#### Speckle Tracking Imaging (STI) and Strain Analysis

**Figure 2:**
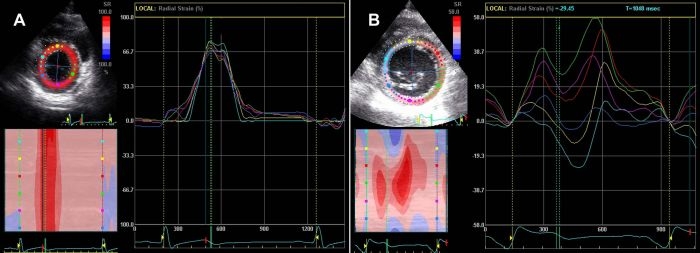
Parametric images and corresponding radial strain tracings. Parametric images and corresponding radial strain tracings on Speckle Tracking Imaging in a representative healthy control (A) and a case with manifest WPW syndrome (B) is shown.

All patients and controls were in sinus rhythm and image acquisition was performed in the resting state in the left lateral position. Standard short axis views at the level of mitral valve were obtained from the left ventricle (LV) at a frame rate of 70 ± 5 frames/second during end-expiratory apnea and stored in cine-loop format for subsequent offline analysis (2.5-MHz transducer Vivid 7, General Electric, Horten, Norway). The endocardial border was then traced using the built-in software at end-systole by a 'point-and-click' approach with care taken to adjust tracking of all endocardial segments. Upon defining this endocardial circle, a larger concentric circle was automatically generated outlining the epicardium with a default width of 11 mm and perimeter adjusted manually as needed to track the myocardium. Strain tracings were then generated using a dedicated software package (EchoPAC™  GE Ultrasound  Systems, GE Vingmed, Horton, Norway) from a  six segment  LV model (Anteroseptal, Anterior, Lateral, Posterior, Inferior and Septal). Radial strain values at peak systolic period (defined as peak segmental radial strain or pRS) were obtained for each individual LV segments using online calipers (Figure 2).

For descriptive purpose, following parameters were calculated: Global LV Peak radial strain = Arithmetic mean of pRS of six individual segments for each case as a surrogate marker of overall LV peak systolic radial strain. To measure heterogeneity, following dispersion indices were calculated (higher dispersion values representing more contractile heterogeneity)): Standard Deviation of pRS (SD ^pRS^): One standard deviation of six  pRS  (corresponding to peak systolic radial strain for each LV segments) in each case. Range of pRS (Range^pRS^): Arithmetic difference between maximum and minimum of six pRS values in each case.

**Figure 3:**
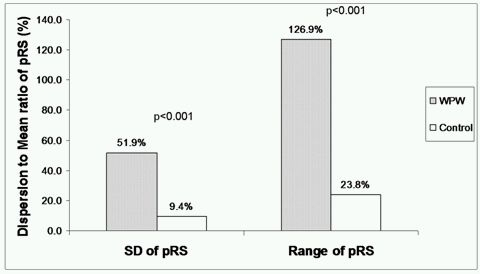
Dispersion indices to mean ratios for WPW and control cohort. Dispersion to mean ratio used a surrogate marker of regional heterogeneity in peak systolic strain pattern of basal left ventricular segments. Abbreviation: SD=Standard deviation, pRS= peak systolic radial strain, WPW=Wolff-Parkinson-White Syndrome.

Finally, ratios of either SD or range to their respective means were displayed as a surrogate marker of overall heterogeneity in systolic strain pattern among basal LV segments (Figure 3).

### Statistical analysis

The data were analyzed using SPSS 13.0 for Windows (SPSS Inc, Chicago, IL, USA). Data are presented as mean ± SD unless stated otherwise. Non-parametric Wilcoxon's signed rank and the Mann-Whitney U test were used to compare the continuous variables in WPW patients and controls. All measurements were done by two independent observers with a minimal degree of variability. Intraclass correlation coefficients (ICC), commonly used as a measure of reliability, with a value of 1 representing a perfect correlation was used to quantify the intraexaminer and interexaminer variation. The ICC was calculated  from mean values according to Portney [18,19].  Two sided p<0.05 was considered statistically significant. ICC values for measured ECHO parameters in our study showed minimal variability (Table 2).

## Results

### Clinical characteristics and pathway localization

The study cohort consisted of eleven cases (7 males, mean age of 34.6 ± 12.0 years) and age-matched eleven controls (6 males, mean age 39.1 ± 8.0 years). Baseline characteristics of the study population and accessory pathway location are shown in Table 1.

### Radial Strain

Parametric images and corresponding radial strain tracings in a control subject and a representative patient with manifest WPW syndrome are shown in Figure 2. No statistically significant difference was observed for mean of pRS (i.e. Global LV Peak radial strain) between WPW and control cohort suggesting overall similar global intensity of radial strain of LV wall. However, dispersion indices of pRS (SD^pRS^ as well as Range^pRS^) were profoundly greater for WPW cases compared to healthy controls (p<0.001) (Table 3). This signifies marked heterogeneity of radial strain among individual LV segments in WPW.  Furthermore, we observed significantly attenuated strain values in segments with overt wall motion abnormalities. Approximately 10.6 % (seven of 66 segments) exhibited marked attenuation of radial strain (≤ 10 %) involving the septum (3 patients), anterior wall (2 patients) and one patient had negative or paradoxical strain (systolic thinning).  However, none of the control patients had any of the above findings.

### Spectral Doppler Analysis and Systolic Time Intervals

As shown in Table 3, PEP and cPEP were significantly longer (p<0.001) in WPW patients when compared with age-matched controls. In contrast, LVET and cLVET values were not significantly different between the two groups. The PEP/LVET ratio was significantly greater (p<0.001) in the WPW group suggesting a depressed state of global systolic function.

## Discussion

Our findings suggest that patients with manifest WPW have evidence of regional contractile dysfunction. This is evidenced from a wide dispersion of pRS {higher SD^pRS^ and RangeSD^pRS^ although the average global LV radial strain was similar to the healthy subjects (mean of pRS)}. Moreover, global systolic contractile function appeared to be depressed as well, as evident from systolic time interval analysis.

### Rationale for using Systolic Time Intervals over Ejection Fraction as a Metric to Assess Global Systolic Function

Ejection fraction (EF), the most frequently used index to assess global cardiac function is the LV stroke volume expressed as a percentage of the end-diastolic volume. This measure has gained popularity largely because it is widely available, easily obtainable and also because no other alternative optimal measures of myocardial contractility have emerged despite extensive investigation. Nevertheless, accurate EF calculation using the recommended Simpsons biplane method requires precise measurement of LV end-diastolic and end-systolic volumes in multiple views with special attention to avoid foreshortening of the LV cavity. Averaged values from both 4-chamber and 2-chamber views, preferably from 2 or 3 beats are recommended for more reliable estimation of ventricular volumes and EF. However, this technique is predicated on geometric assumptions (i.e. extrapolation of 2-dimensional data to 3-dimensional volume) and although it is fairly accurate in ventricles with symmetric contractility, it is less reliable in ventricles with overt/subtle regional wall motion abnormalities or geometrically deformed ventricles such as ventricular aneurysms. This limitation of the Simpsons method to reflect regional asymmetry of LV function is the reason why EF was not selected as the preferred metric to assess global systolic function in the present study.

### Wall motion abnormalities and contractility

Systolic and diastolic 'wall motion' abnormalities have been well described  in patients with WPW Syndrome [1-3,5,20].  The interventricular septal abnormalities  in the latter are quite similar to those observed in LBBB. The normalization of  interventricular septal motion with normalization of QRS complex (by pacing techniques) in type B WPW syndrome strongly suggest that the abnormal motion is related to the altered  sequence of ventricular depolarization during pre-excitation [21]. This concept is further supported by the normalization of radial strain patterns in one of our patients with intermittent preexcitation, when non pre-excited beats were analyzed (data not shown). A similar observation was made by other investigators who reported resolution of abnormal interventricular septal motion (in nonpreexcited beats) in patients with intermittent ventricular pre-excitation [5,21].

In our study, WPW cases exhibited a wide dispersion in pRS values of LV segments during systole suggesting markedly heterogenous strain pattern and contractility. It has to be emphasized that wall motion abnormalities are not necessarily always associated with altered contractility. Toyoda et al studied interventricular septal (IVS) contractile patterns in patients post coronary artery bypass surgery (CABG) and reported that peak tissue Doppler velocities in the IVS were attenuated [6]. However, there was no observed difference between peak systolic strain or strain rates in patients post CABG  compared to the non- CABG group [6]. This is particularly of interest as  numerous experimental and clinical studies in the setting of coronary artery disease [22-24] have shown the superiority of strain analysis over tissue Doppler in discerning contractility from  tethering and translation.

Radial strain data obtained in our patients extends the well-recognized observations of pre-excitation on wall motion and unequivocally demonstrates its detrimental effects on regional left ventricular function.

### Possible underlying mechanisms for contractile dysfunction

The transmural arrangement of myocardial fibers is not uniform across the wall of the LV. Subendocardial and subepicardial muscle bundles are aligned longitudinally (long axis contraction), with a slight spiral arrangement, and midwall fibers are aligned circumferentially (radial axis contraction). Although the precise mechanism for abnormal contractile function in the pre-excited state remains speculative, we believe that accessory pathway conduction leads to ventricular depolarization via myocyte-myocyte conduction as opposed to the normal orderly propagation of the impulse via the His-Purkinje system. Depending on the degree of pre-excitation, ventricular insertion site of bypass tract and/or layer of muscle fibers/bundles being recruited, further heterogeneity of depolarization may ensue. As observed with dependent right ventricular apical pacing [13] it is plausible that the 'location' of the bypass tract or 'chronicity' of preexcitation could further influence pattern and extent of systolic dysfunction.

### Systolic Time Intervals and Global LV Performance

Systolic time intervals have been reported as reliable surrogate parameters of left ventricular systolic function. Electromechanical systole comprises the PEP and LVET. LVET is the effective contraction phase during electromechanical systole. Weissler and co-workers [16-18] have emphasized the use of the PEP/LVET ratio as a very sensitive index of ventricular function. A recent publication by Baker and others studied the effect of Cardiac Resynchronization Therapy on systolic time intervals [25]. They reported a decrease (improvement) in the PEP/LVET ratio, a time measure of LV systolic performance. These investigators ascribed PEP shortening post-CRT, to a reduction in the electromechanical delay (time from onset of ventricular depolarization to ventricular contraction). We observed a significant prolongation of PEP without a significant change in LVET in the WPW group, representing significant electromechanical delay despite early depolarization or ventricular preexcitation. It is tempting to speculate that the resulting prolongation of electromechanical systole (onset of depolarization to aortic valve closure) could plausibly truncate diastolic filling time, further compromising cardiac function. Emmel et al reported a case series of four children (six-year follow up) with cardiomyopathy and preexcitation (right-sided pathways) in the absence of sustained tachyarrhythmia; three of these patients had complete resolution of left ventricular dysfunction following accessory pathway ablation. These compelling observations complement our data and confirm that dilated cardiomyopathy of a reversible nature may be associated with manifest preexcitation  [26].

### 2D Strain/ Speckle Tracking Imaging and Regional LV function

During the cardiac cycle, regional deformation of the myocardium occurs in three major directions: longitudinally, circumferentially and radially, all of which can be quantified using 2-dimensional strain techniques providing real-time automatic assessment of regional cardiac function. This technique analyzes myocardial motion objectively by tracking natural acoustic markers in grey scale ultrasonic images in two dimensions (speckle tracking) and is a validated Doppler-independent method of quantifying regional cardiac deformation [10,11,27]. The concept is similar to magnetic tags in MRI, but acoustic tags are more persistent than magnetic tags and can be analyzed during the entire cardiac cycle and also reveal beat-to-beat variability. Speckle Tracking Imaging is less angle dependent than Doppler techniques and allows circumferential and radial assessments of deformation otherwise not possible with Doppler-based imaging. It has higher temporal and spatial resolution compared to MRI-tagging techniques. In addition, it is less time consuming, less expensive and has enhanced practical applications [28-31]. Serri and colleagues recently used 2D strain (STI) and demonstrated a significant attenuation of radial, longitudinal and circumferential strain in patients with hypertrophic cardiomyopathy [29. Radial strain was useful in differentiating transmural from nontransmural infarctions with fairly high specificity in a recent study by Becker and colleagues [28].  More  recently radial strain timing parameters were used to quantify dyssynchrony and predict response to cardiac resynchronization therapy [31]. It is therefore clear that strain-based techniques are able to accurately characterize tissue deformation and that strain estimates can be conceived as being analogous to regional ejection fraction [32].

We found the dispersion indices like standard deviation or range segmental radial strain to be superior estimate of heterogeneity of regional contractility compared to mean radial strain. Interestingly, we also observed hypercontractility in segments diametrically opposite to those with impaired radial strain probably accounting for the lack of difference in mean radial strain values and ejection fraction between groups.

### Clinical Implications

Abnormal motion does not always imply contractile dysfunction. With 2D strain imaging, an angle-independent quantification of regional cardiac deformation, relatively independent of wall motion is possible. Our data suggests that the preexcited state is associated with an impairment of regional and global left ventricular contractility. In some respects, the mechanism by which RV apical pacing or left  bundle branch block adversely influence LV contractility in the setting of congestive heart failure appear to be similar to those responsible for impaired contractility in the pre-excited state; both essentially mediated by  electromechanical delay. The extent, progression and reversibility of left ventricular dysfunction in the setting of  chronic  preexcitation  however, is unknown but  certainly merits further study.

### Study Limitations

The number of patients enrolled was limited and they predominantly appeared to have right-sided pathways, even though we enrolled consecutive patients; whether or not patients with left sided pathways have disordered contractility is uncertain. The accuracy of pathway localization using the surface ECG has inherent drawbacks; clearly localization of pathways by electrophysiological studies would be desirable. Speckle Tracking Imaging is very image quality dependent and short axis images cannot eliminate longitudinal motion entirely. We found frame rates in the range of 65 Hz  to be most suitable for speckle-tracking analysis and the algorithm performed poorly with frame-rates of > 100 Hz.

We used a modified method for systolic time interval assessment using Doppler-based data but had a control group for comparison; however, direct comparison of normative values with established techniques would have to be done with caution. Controversy exists regarding 'rate' correction for systolic time intervals; it is also not clear whether the prolonged PEP intervals ought to be corrected in the setting of LV conduction blocks. No specific recommendations relating to PEP adjustments in patients with pre-excitation could be found in the literature.

## Conclusion

Our observations indicate that patients with manifest WPW (predominantly right-sided pathways) have impairment of regional contractility as ascertained by radial strain dispersion parameters, resulting from the abnormal temporal sequence of activation in the pre-excited state.  Differences in surrogate measures of global contractility such as PEP/LVET seem to indicate global LV dysfunction in this cohort of patients. Our findings will need to be validated in a larger group of patients that includes left-sided pathways.

## Figures and Tables

**Table 1:**
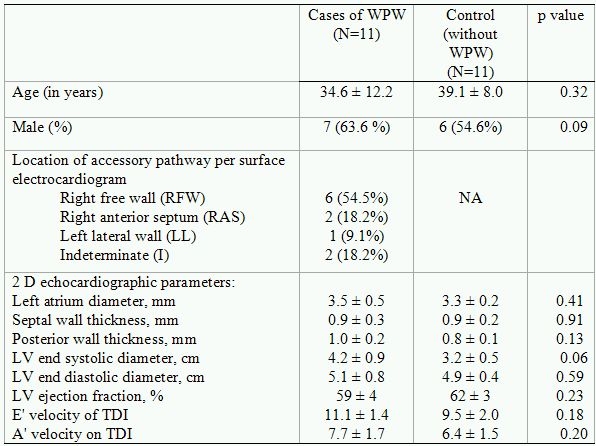
Study cohort characteristics

Data presented as Mean ± Standard deviation or number (frequency) N=Number of patients, WPW=Wolff-Parkinson-White syndrome, LV=Left ventricle, TDI=Tissue Doppler imaging

**Table 2:**
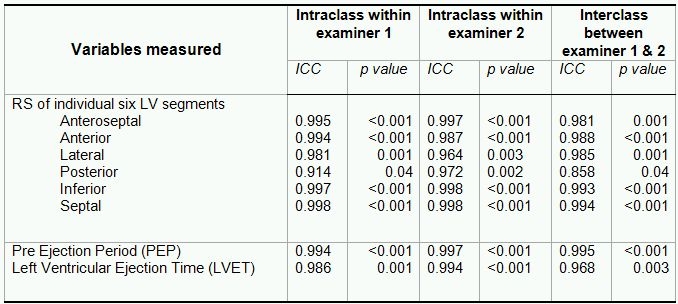
Interclass Correlation Coefficients (ICC) for variables measurement within and between two examiners for the study cohort

RS=Peak radial strain, LV=Left Ventricluar, ICC=Interclass correlation coefficient

**Table 3:**
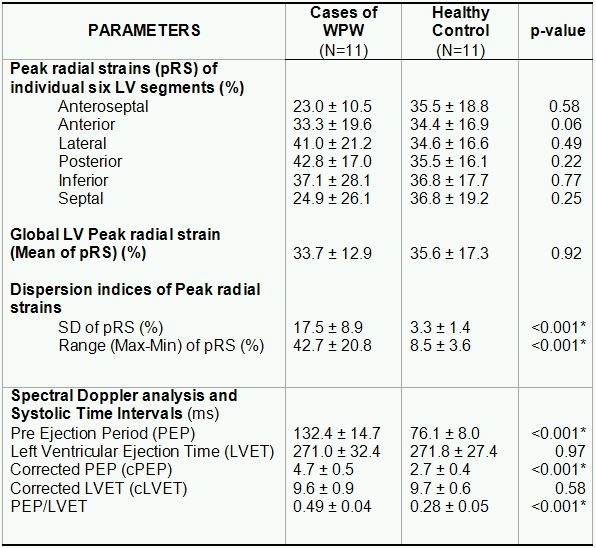
Strain pattern and systolic time interval parameters of study cohort

N=Number of patients, WPW=Wolff-Parkinson-White syndrome, LV=Left ventricle, SD=Standard Deviation, Max=Maximum, Min=Minimum, ms=millisecond. Data are presented as Mean ± Standard deviation or number (frequency)
